# 

**Published:** 1841-12

**Authors:** 


Volume II. DECEMBER, 1841. No. 2.

THE

AMERICAN

### JOURNAL AND LIBRARY

OF

### Dental Science.

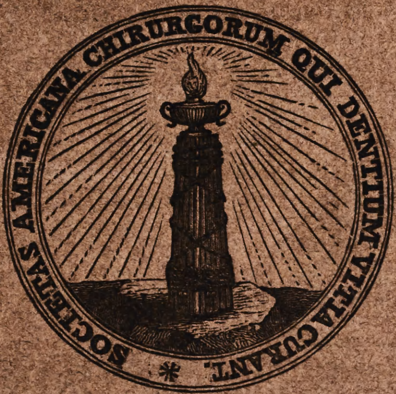

PUBLISHED QUARTERLY.

PUBLISHED UNDER THE AUSPICES OF THE

**American Society of Dental Surgeons.**

EDITED BY

CHAPIN A. HARRIS, M. D., BALTIMORE,

AND

SOLYMAN BROWN, M. D., NEW YORK.

NEW YORK:—GEORGE ADLARD,

BALTIMORE:—ARMSTRONG & BERRY,

LONDON:—SAMUEL HIGHLEY.

WOODS AND CRANE, PRS., BALT.

**$ 5 00 per annum, payable in advance.**

This number contains 10 sheets.

